# Morphometric Anatomy of the Tibia Plateau in Nigerians

**DOI:** 10.4314/ejhs.v32i1.17

**Published:** 2022-01

**Authors:** Dennis Erhisenebe O Eboh

**Affiliations:** 1 Department of Human Anatomy and Cell Biology, Faculty of Basic Medical Sciences, College of Health Sciences, Delta State University, Abraka, Nigeria

**Keywords:** Arthroplasty, forensic anthropology, knee joint, orthopedics, Tibia, Nigeria

## Abstract

**Background:**

There are increasing cases of osteoarthritis and associated disabilities with age, hence the need for knee replacement to restore the anatomy and function of the knee. The objective of this study was to determine the dimensions of the tibial plateau in dry human tibias of Nigerians. This will serve as a guide for the tibia components during total knee replacement procedure.

**Methods:**

This study adopted the descriptive method of the quantitative design and utilized 133 dry tibias. Total transverse width, total anteroposterior length of intercondylar region, transverse widths of medial and lateral tibia plateaus, and anteroposterior length of the plateaus were measured in millimeter (mm). Statistical analysis of the data was done using mean (SD), t-test and correlation, with the aid of SPSS 23. Statistical significance was fixed at p<0.05.

**Results:**

Statistically, the differences between the right and left parameters were not significant. The mean total transverse width was longer than the total anteroposterior length. The mean anteroposterior length of the medial tibia plateau was significantly longer than that of the lateral tibia plateau. The difference between the transverse width of both the medial and lateral tibia plateau was not statistically significant.

**Conclusion:**

This study showed that the tibial plateau dimensions can act as guiding tools to the orthopedic surgeon during a knee replacement procedure; and those involved in the fabrication of knee replacement prostheses for Nigerians. The physical anthropologists will also find the anthropometric data invaluable in population studies.

## Introduction

The tibia plateau or condyle is the upper articular surface of the tibia. It slopes posteriorly and downwards which decreases with age from birth; and it is more prominent in people who are habitual squatters (1). The plateau is formed into a two concave particular area; the medial condyle which is oval anteroposteriorly and lateral condyle which is almost circular and slightly smaller than the former. They articulate with the corresponding femoral condyles to form part of the knee joint. An anteroposterior groove separates the intercondylar eminence to form the medial and lateral intercondylar tubercles) (2).

In view of the increasing cases of osteoarthritis and the attendant disability (3) with age (4), and the need for knee replacement to restore the anatomy and function of the knee (5), a population-specific data of the proximal tibia become crucial. This is so because the dimensions of the superior articular surface of the tibia have been found to differ among populations (6,7). It has also been stated that variations in anthropometry exist among different populations of the world (8). These parameters have been well studied in Caucasian, Indian and East Asian subjects, but there is a dearth of information about the proximal tibia anthropometric parameters of the black African population. In a revision of 30 studies, only 3 of them, including 130 knees, belonged to black subjects of which none is African (9). Also, different anatomical profiles would not fit correctly with conventional components of knee prosthesis (10).

The linear dimensions of the tibia plateau had been done utilizing dry tibia bones (direct method) (8,11–13), computed tomography (CT) (14–16), and magnetic resonance imagining (MRI) (17,18). Despite the availability of studies on tibia plateau in the literature, there is a paucity of same in Nigerians. This study tends to fill the gap using the tibial plateau dimensions of Nigerians.

The basic morphometric data from this study will be very important to the orthopedic surgeons in planning for knee replacement procedures, and prosthetic industries involved in production of knee replacement prostheses. These primary data will also be of immense benefit to the anthropologist in comparing the tibia condyles with those of other ethnic groups or populations. The purpose of this study was to determine the dimensions of the tibia plateau in dry human tibias of Nigerians.

## Materials and Methods

The study used the descriptive method of the quantitative design. All dry human tibias in the anatomy museum of five Nigerian Universities: Ambrose Ali University, Delta State University, Nnamdi Azikiwe University and University of Benin, formed the study population. One hundred and thirty-three (133) tibias constituted the sample for the study, which amounted to all the available tibias in the bone collections. The Faculty Ethics Committee of the Delta State University, approved the research (REC/FBMS/DELSU/19/47), without any prejudice to the Declaration of Helsinki as revised (19).

**Figure 1:**
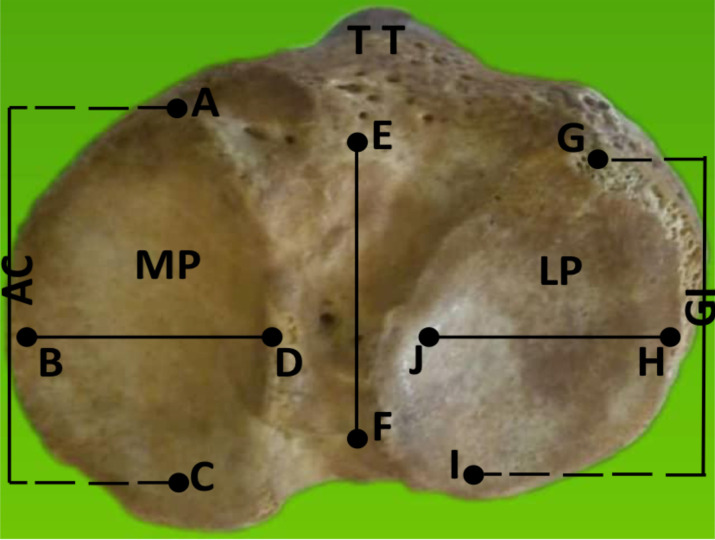
Proximal tibia with all measurements taken. AC: Length of medial tibial plateau; BD: Width of medial tibial plateau; GI: Length of lateral tibial plateau; JH: Width of lateral tibial plateau; DJ: intercondylar eminence; EF: Total anteroposterior length of tibia plateau; BH: Total transverse width of tibia plateau; MP: Medial tibial plateau; LP: Lateral tibial plateau; TT: Tibial tuberosity.

**Method of measurements**: Firstly, the bones were separated into right and left based on anatomical position. All bones with normal morphological features were involved in the study, while those with abnormal features and fractures were excluded. All the bones were adults of unknown gender. The following parameters were measured in millimeter according to Figure 1, using the digital sliding caliper (Mitutoyo, Japan): total transverse width (TTW) of the tibia plateau, total anteroposterior length (TAL) of the intercondylar region, the transverse width of the lateral tibia plateau (TWL), the anteroposterior length of the lateral plateau (ALL), the transverse width of the medial tibia plateau (TWM) and the anteroposterior length of the medial plateau (ALM).

**Data analysis**: Analysis of the data was done with the aid of IBM SPSS statistic 23. Mean and standard deviation were used to summarize the data, while independent (unpaired) samples t-test was used to test if there was a significant mean difference between data on the right and left sides, and paired samples t-test between the medial and lateral sides. All p-values <0.05 were regarded statistically significant.

## Results

Results showed that of the 133 dry tibias used for the study, 51.1% (68) were from the right and 48.9% (65) from the left. Table 1 showed results of independent samples t-tests that in all the parameters measured, the differences between the right and left sides were not significant statistically (p>0.05), as well as the mean dimensions of combined data on both sides. The mean total transverse width was greater when compared to the total anteroposterior length.

**Table 1:** Comparison of Tibia plateau parameters measured between right and left sides

Parameter	Data	Range	Mean (SD)	t	p-value
Anteroposterior length of medial tibia	Total	28.36–53.79	44.36(4.96)	-	-
plateau (mm)	Right	29.27–52.46	45.03(4.23)	1.592	0.114
	Left	28.36–53.79	43.66(5.58)		
Transverse width of medial tibia	Total	21.86–11.36	30.93(3.40)	-	-
plateau (mm)	Right	21.86–11.36	31.38(3.12)	1.559	0.121
	Left	23.58–37.36	30.47(3.64)		
Anteroposterior length of lateral tibia	Total	22.06–18.37	39.4394(4.87)	-	-
plateau (mm)	Right	22.06–18.15	40.1029(4.41)	1.609	0.110
	Left	23.19–18.37	38.7452(5.26)		
Transverse width of lateral tibia	Total	18.36–10.59	31.36(3.89)	-	-
plateau (mm)	Right	18.36–37.14	31.7372(3.70)	1.150	0.252
	Left	21.44–10.59	30.9628(4.06)		
Total transverse width of tibia	Total	31.99–81.68	70.37(7.49)	-	-
plateau (mm)	Right	42.31–81.68	71.1569(6.76)	1.244	0.216
	Left	31.99–79.90	69.5440(8.15)		
Total anteroposterior length of tibia	Total	28.24–71.95	45.82(5.69)	-	-
plateau (mm)	Right	28.24–52.19	45.9824(4.51)	0.328	0.743
	Left	28.56–71.95	45.6571(6.75)		

SD: Standard deviation

Results of paired samples t-test showed that the mean anteroposterior length of the medial plateau was longer than that of the lateral plateau and the difference was statistically significant (p<0.001; t=19.916). However, paired samples t-test also showed that the difference between the transverse width of the medial plateau and that of the lateral plateau was not statistically significant (p=0.067; t= -1.850).

Table 2 shows the comparison of the mean dimensions of the tibia plateaus in the present study and those of other populations. The mean total transverse width of the tibia plateau in the present study is greater than those of the Kenyan and Thai populations, but lower than those of South East Nigerian and Iranian populations. However, the mean total anteroposterior length of the tibia plateau in the present study is lower than those of the Kenyan, South East Nigerian and Iranian populations, but similar to that of the Thai population.

**Table 2:** Comparison of mean dimensions of tibia plateau of present study and those of other populations

Dimension (mm)	Data	Population/ Mean (SD)							

		South Indian (8)	Kenyan (11)	South East Nigerian (13)	Iranian (14)	Thai (18)	North Indian (22)	French (23)	Present study (Nigerian)
Anteroposterior length of medial tibia plateau	Total	39.80(3.38)	42.06	-	50.50(4.39)	-	-	50.80 (3.30)	44.36(4.96)
	Right	40.60(3.90)	42.28	-	-	-	45.42(4.17)*		44.03(4.23)
	Left	39.20(3.60)	41.83	-	-	-	45.05(4.51)*	-	43.66(5.58)
Transverse width of medial tibia plateau	Total	26.70(2.80)	27.21	-	-	-	-	-	30.93(3.40)
	Right	26.90(2.90)	27.13	-	-	-	28.72(2.94)*	-	31.38(3.12)
	Left	26.60(2.70)	27.29	-	-	-	28.17(2.66)*	-	30.47(3.64)
Anteroposterior length of lateral tibia plateau	Total	33.60(3.70)	37.43	-	48.90 (5.0)	-	-	47.20 (3.30)	39.44(4.87)
	Right	34.80(3.70)	38.17	-	-	-	38.82 (2.81)*		40.10(4.41)
	Left	32.60(3.40)	38.68	-	-	-	39.00 (3.97)*	-	38.75(5.26)
Transverse width of lateral tibia plateau	Total	26.10(2.90)	26.78	-	-	-		-	31.36(3.89)
	Right	26.5 (3.4)	26.96	-	-	-	27.33(2.81)*	-	31.74(3.70)
	Left	25.70(2.50)	26.61	-	-	-	27.41(3.09)*	-	30.96(4.06)
Total transverse width of tibia plateau	Total	-	69.38	75.30(0.67)	74.60 (5.9)	68.80(5.80)	-	-	70.37 (7.49)
	Right	-	70.09	-	-	-	-	-	71.16(6.76)
	Left	-	68.66	-	-	-	-	-	69.54(8.15)
Total anteroposterior length of tibia plateau	Total	-	49.38	55.00(5.56)	48.60 (4.5)	46.04(4.40)	-	-	45.82(5.69)
	Right	-	49.99	-	-	-	-	-	45.98(4.51)
	Left	-	48.77	-	-	-	-	-	45.66(6.75)
**Method used**		Direct	Direct	Direct	MRI	MRI	Direct	CT scan	Direct

* Average of mean values for male and female

## Discussion

In this study, the mean total transverse width was almost similar to that of Lakati and Ndeleva (11) in Kenya, but greater than that of Gupta et al. (10) in South India. The mean total transverse width in the current study was also similar to the average of the combined male and female data on both sides in a study conducted in Bihar region, India, by Sinha and Prasad (20). Moghtadaei et al. (14) in Iran also conducted a similar study and the mean total transverse width was greater than that of the present study. The reason for this difference could be because the former used CT scan as against the direct method used in this study. Previous studies have shown that the direct method was more accurate than the radiological methods (8, 21).

The mean total transverse width of the present study is slightly lower than the average of the mean total transverse width of males and females in a study by Fan et al. (17) who studied the tibia plateau in Southeastern China, using the Magnetic Resonance Imaging scans (MRI). A similar study in South-East Nigeria (13) reported a mean total transverse width which is greater than that of the present study; and this can be attributed to differences is sample size.

The anteroposterior length of the tibia plateau in the current study was similar in dimension to that of Gupta et al. (10) in South India. It is, however, lower than those reported by Fan et al. (17), Moghtadaei et al. (14) and Lakati and Ndeleva (11) and Katchy et al (13). This may be due to factors such as error of measurements, different methods adopted which affect anthropometric measurements, ethnic differences due to geographic locations and differences in sample size.

Comparing the anteroposterior and transverse dimensions of medial and lateral plateaus, the results of the present study showed that the anteroposterior length of the medial plateau was greater than that of the lateral one. This is important, especially for prosthetic designs that puts into consideration the difference in medial and lateral plateau in order to eliminate the problem of asymmetric design. A similar report on the difference in anteroposterior dimension between medial and lateral plateaus had been made in previous studies (22,23). Nonetheless, the transverse width of both plateaus was approximately equal in dimension in the present study.

The observation on anteroposterior and transverse dimensions of the plateaus was similar to that of Gupta et al. (10) in a study of 50 dry tibias in South India population. Similarly, another study that utilized 52 dry tibia bones in Kenya, by Lakati and Ndeleva (11), showed that the mean combined anteroposterior dimension was higher in medial plateau than the lateral plateau, while the mean transverse dimension was equal in both plateaus. Srivastava et al. (24) also studied 150 dry tibias of North Indian subjects and the result showed that the anteroposterior dimension was higher in the medial plateau than the lateral plateau, while the transverse dimension was equal in both plateaus. Sinha and Prasad (20) conducted a study using 50 dry bones in Patna, Bihar, India, and the results revealed that both the anteroposterior and transverse dimensions were higher in the medial than the lateral plateau. Gandhi et al. (22) conducted a similar study using 100 dry tibia bones in North India, and the analysis of the result revealed that the average of the mean combined anteroposterior length and transverse width of males and females were higher in medial plateau than in lateral plateau. In a study that used 50 dry tibia bones in Chennai, conducted by Shree et al. (12), the average of mean right and left anteroposterior dimension was slightly higher in the medial plateau than the lateral plateau; but the reverse was the case for the transverse dimensions. In some previous studies, like those of Prasanna et al. (25) and Murlimanju et al. (8), they observed that both the anteroposterior and transverse dimensions were statistically higher in the medial plateau compared to the lateral plateau. In other studies, by Lucena et al. (26) and Fan et al. (17), they reported that the anteroposterior dimension in the medial plateau was greater than that of the lateral plateau, but in contrast, the transverse dimension of the lateral plateau was greater than that of the medial one.

The variation observed between the present and previous studies could be ascribed to environmental factors, genetic factors, difference in the geographic location of the various populations studied, and the different methods that might have been used in the different studies which can affect anthropometric measurements.

The implication of the variability of Knee anatomy between contemporary Nigeria population and those of European, American, Asians, and so forth, is that different anatomical profiles would not fit correctly with conventional components of knee prosthesis that are designed for Caucasians and Americans. Therefore, prostheses specific for the Nigeria population is necessary to avoid the problem of under-sizing and oversizing tibial components in total knee arthroplasty.

The data in this present study were not categorized into males and females, hence no dimorphic assessment was done; this is a limitation of the study. It is therefore suggested that a morphometric radiographic assessment of tibia plateau, with a focus on gender dimorphism should be conducted in future time. Furthermore, this study used only dry bones; hence it is suggested that studies involving living tissues be conducted for comparison.

Based on the results of the present study, it can be concluded that the total transverse width of the tibia condyle was longer than the anteroposterior length and the medial tibia plateau was longer than the lateral plateau. Furthermore, there was no significant difference in dimensions between the transverse widths of both plateau and also between corresponding dimensions on both sides. The data documented in the present study will be useful to the orthopedic surgeon in selecting prostheses of suitable sizes, and the manufacturing industry of tibia prostheses for the Nigeria population. They will also be useful to the physical anthropologists in population studies.
